# Supplementary material for: p53-Mediated Radiosensitization of 177Lu-DOTATATE in Neuroblastoma Tumor Spheroids
Source: Biomolecules. 2021 Nov 15;11(11):1695. doi: 10.3390/biom11111695 (PMC8615514; doi:10.3390/biom11111695)
Supplement: Supplementary file 1 [file biomolecules-11-01695-s001.zip › biomolecules-1421580-supplementary/Supplementary data/FigureS1_HPLC.pdf]

(a)

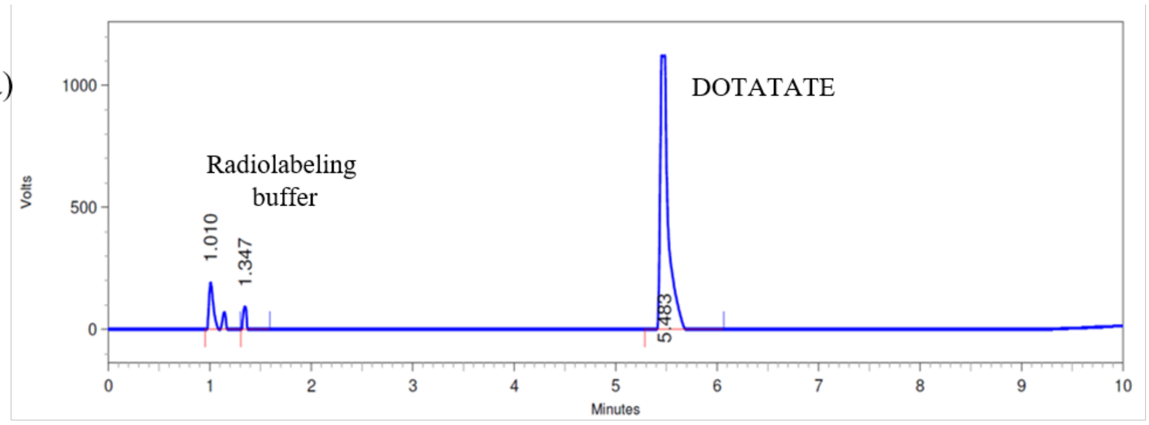


**AID-UV Results**

| Retention Time | Area | Area % | Height | Height % |
|---|---|---|---|---|
| 1.010 | 2839063 | 10.10 | 753569 | 13.46 |
| 1.347 | 798521 | 2.84 | 364161 | 6.51 |
| 5.483 | 24474604 | 87.06 | 4479014 | 80.03 |
| Totals | 28112188 | 100.00 | 5596744 | 100.00 |

(b)

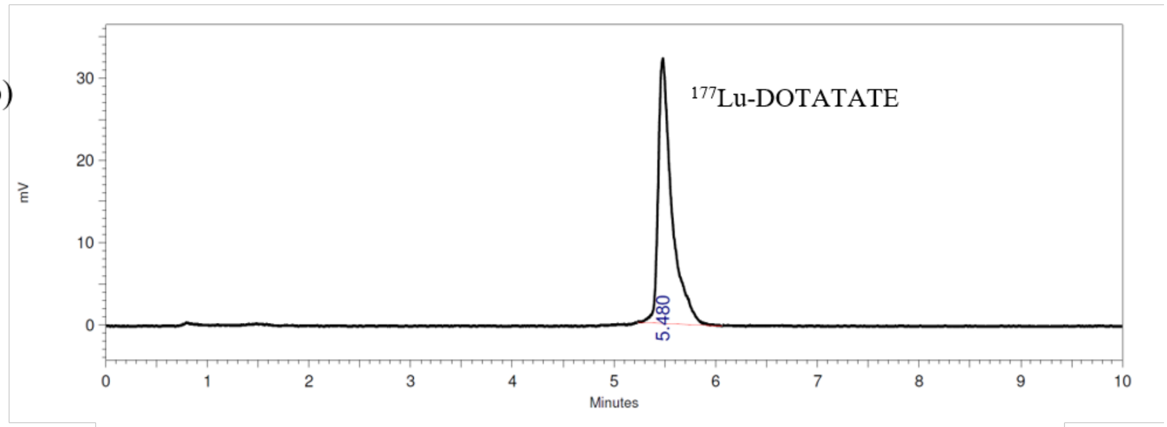


**AID-Radio Results**

| Retention Time | Area | Area % | Height | Height % |
|---|---|---|---|---|
| 5.480 | 1200405 | 100.00 | 128938 | 100.00 |
| Totals | 1200405 | 100.00 | 128938 | 100.00 |

Figure S1: HPLC chromatograms of (a) commercial DOTATATE (UV-detector) and (b) $^{177}$Lu-DOTATATE (Radio-detector).
