# Supplementary material for: p53-Mediated Radiosensitization of 177Lu-DOTATATE in Neuroblastoma Tumor Spheroids
Source: Biomolecules. 2021 Nov 15;11(11):1695. doi: 10.3390/biom11111695 (PMC8615514; doi:10.3390/biom11111695)
Supplement: Supplementary file 1 [file biomolecules-11-01695-s001.zip › biomolecules-1421580-supplementary/Supplementary data/FigureS2_labeling stability.pdf]

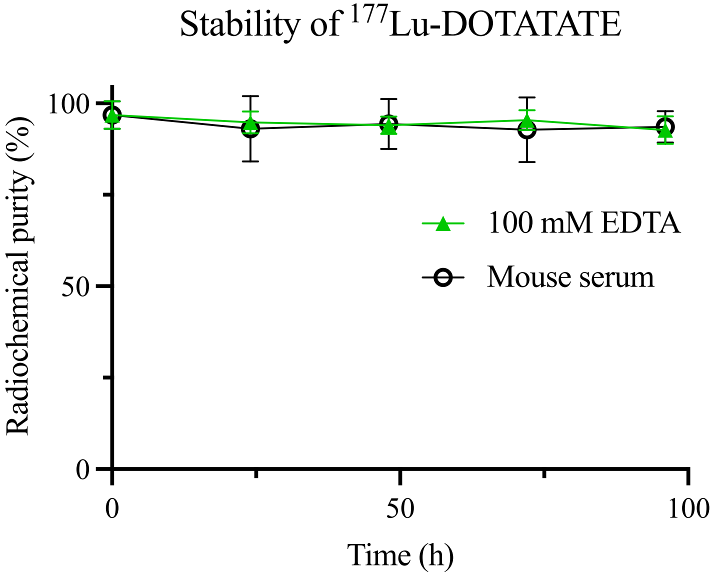

Figure S2: Stability of ^{177}Lu-DOTATATE in the presence of 100 mM EDTA or mouse serum (mean±SD, N=3).
