# Supplementary material for: p53-Mediated Radiosensitization of 177Lu-DOTATATE in Neuroblastoma Tumor Spheroids
Source: Biomolecules. 2021 Nov 15;11(11):1695. doi: 10.3390/biom11111695 (PMC8615514; doi:10.3390/biom11111695)
Supplement: Supplementary file 1 [file biomolecules-11-01695-s001.zip › biomolecules-1421580-supplementary/Supplementary data/FigureS3 - XTT + spheroid ctrl_v9.pdf]

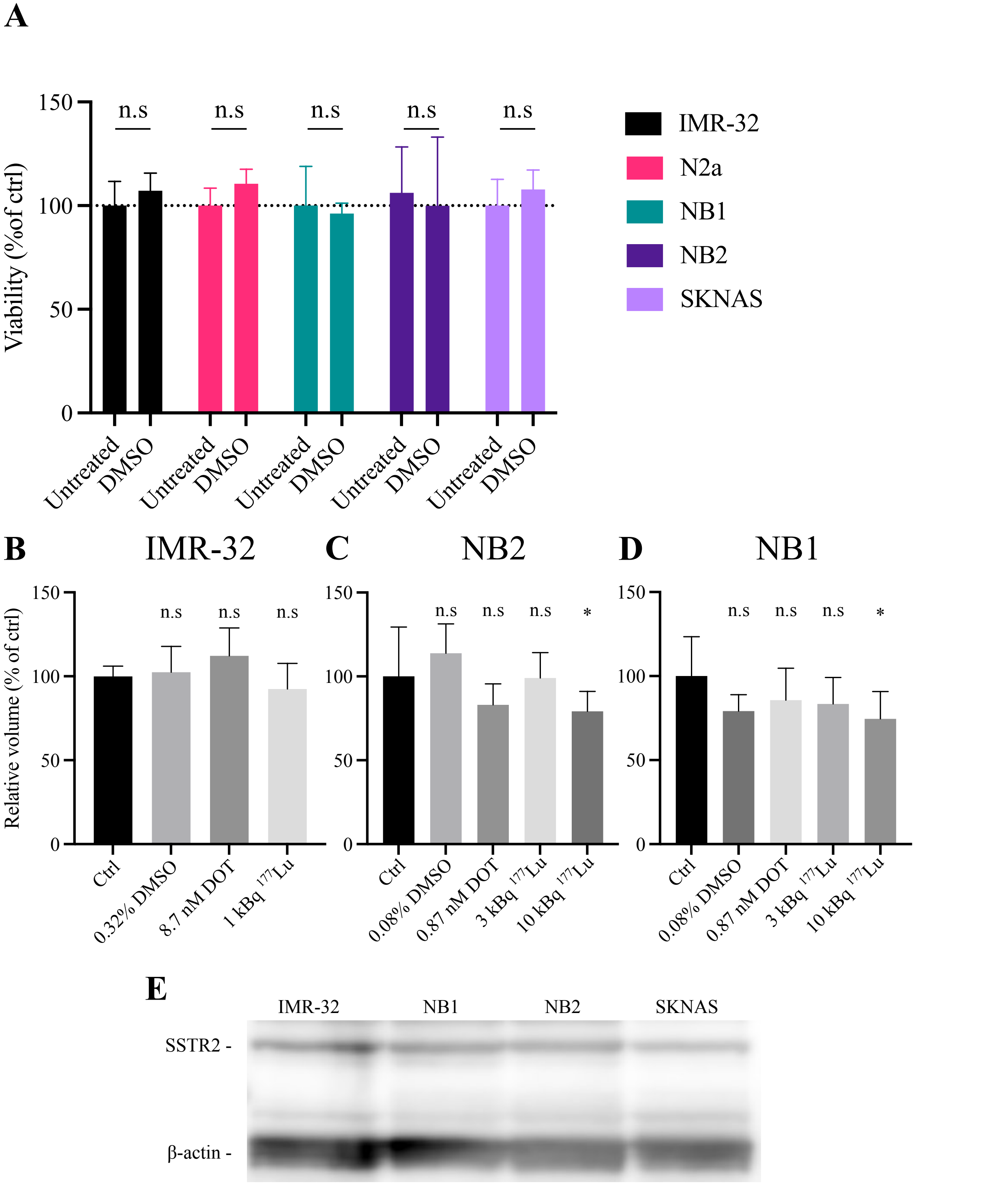

Figure S3:XTT and spheroid controls. (a) Viability of cells treated with DMSO corresponding to the highest concentration of VIP116 for each cell line. (b-d) Spheroid volume at the last investigated time point (% of untreated control) of spheroids treated with DMSO, unlabeled DOTATATE (DOT) and ^{177}Lu for (b) IMR-32, (c) NB2 and (d) NB1. 0.08% DMSO corresponded to the same DMSO content as present in 8 μM VIP116 and 0.32% corresponded to 32 μM. 0.87 nM DOTATATE corresponded to the same molar concentration DOTATATE as present in 10 kBq ^{177}Lu-DOTATATE and 8.7 nM corresponded to 100 kBq. (e) Representative western blot of SSTR2 in untreated IMR-32, NB1, NB2 and SKNAS lysates.
