# Supplementary material for: p53-Mediated Radiosensitization of 177Lu-DOTATATE in Neuroblastoma Tumor Spheroids
Source: Biomolecules. 2021 Nov 15;11(11):1695. doi: 10.3390/biom11111695 (PMC8615514; doi:10.3390/biom11111695)
Supplement: Supplementary file 1 [file biomolecules-11-01695-s001.zip › biomolecules-1421580-supplementary/Supplementary data/FigureS4_HCT116_v2.pdf]

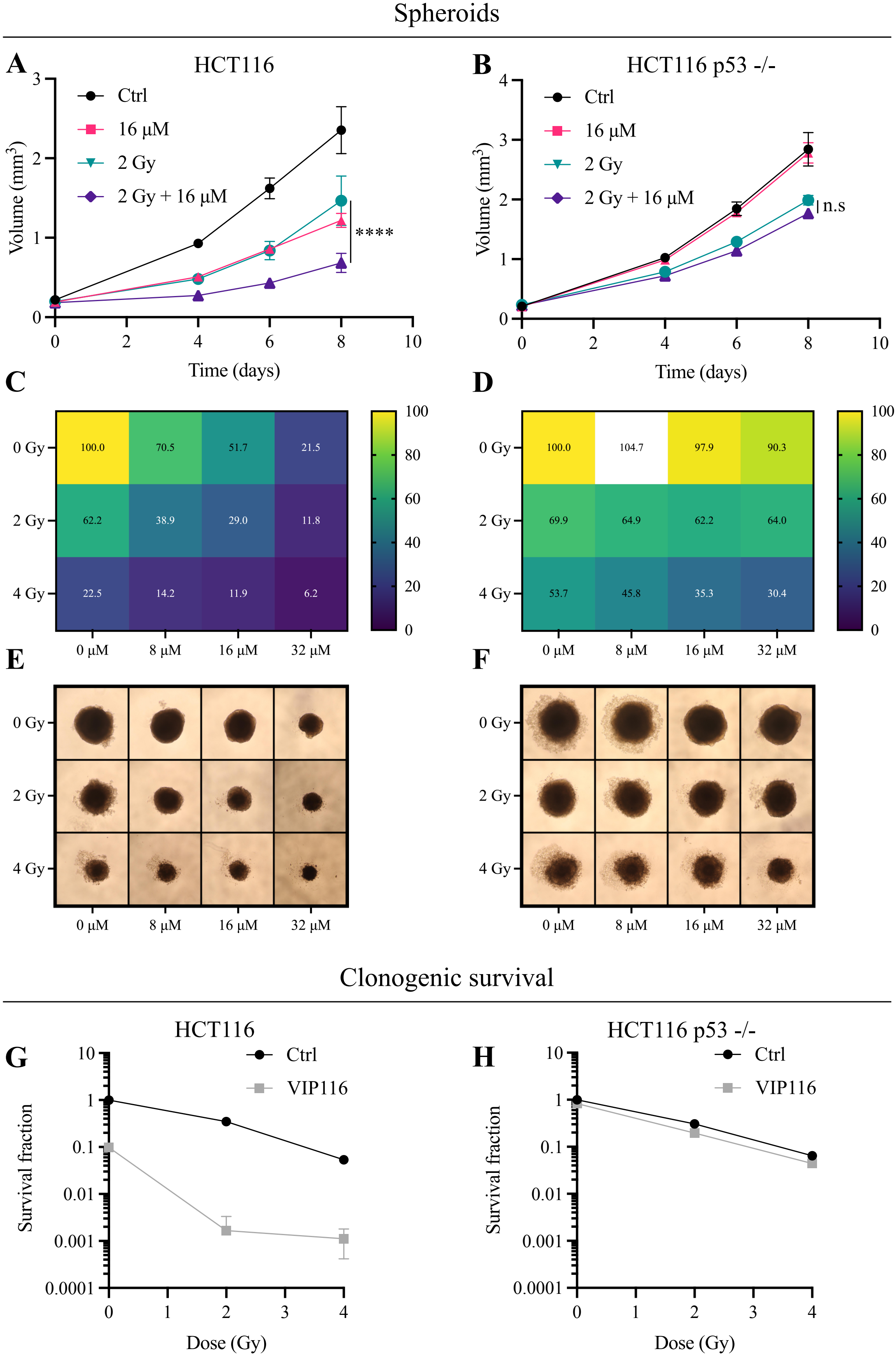

Figure S4: Specificity of VIP116. (a-f) Spheroids treated with VIP116 and/or external beam radiation on (a, c, e) HCT116 with wtp53 or (b, d, f) HCT116 p53 knock-out. (a-b) Representative graphs of (a) HCT116 or (b) HCT116 p53-/- spheroids treated with 16 µM VIP116 and/or 2 Gy radiation (mean±SD, N=3). (c-d) Spheroid size (% of untreated control) of (c) HCT116 or (d) HCT116 p53-/- at day 8. (e-f) Representative (e) HCT116 or (f) HCT116 p53-/- spheroid images at day 8. (g-h) Clonogenic survival of (g) HCT116 or (h) HCT116 p53-/- treated with 0-4 Gy radiation and/or 16 µM VIP116 (mean±SD, N=3).
