# Supplementary material for: p53-Mediated Radiosensitization of 177Lu-DOTATATE in Neuroblastoma Tumor Spheroids
Source: Biomolecules. 2021 Nov 15;11(11):1695. doi: 10.3390/biom11111695 (PMC8615514; doi:10.3390/biom11111695)
Supplement: Supplementary file 1 [file biomolecules-11-01695-s001.zip › biomolecules-1421580-supplementary/Supplementary data/FigureS5 - spheroid combination_v3.pdf]

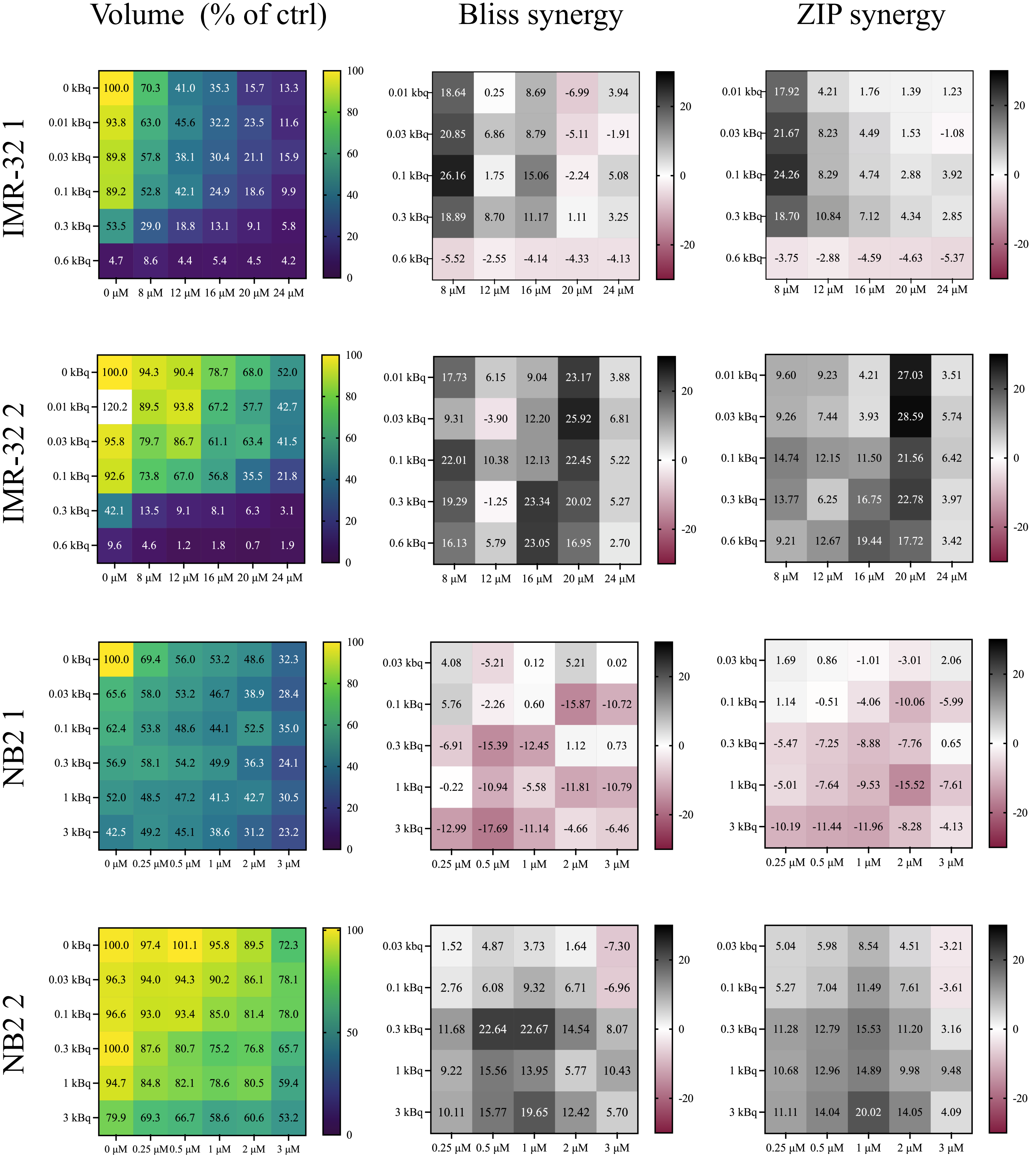

Figure S5: Spheroid combination therapy replicates for IMR-32 (1st and 2nd row) and NB2 (3rd and 4th row). Left panel: Average spheroid volume (% of ctrl) at last time point. Middle and left panel: synergy values for each combination, calculated with Bliss (middle panel) or ZIP (left panel) models.
