# Supplementary material for: p53-Mediated Radiosensitization of 177Lu-DOTATATE in Neuroblastoma Tumor Spheroids
Source: Biomolecules. 2021 Nov 15;11(11):1695. doi: 10.3390/biom11111695 (PMC8615514; doi:10.3390/biom11111695)
Supplement: Supplementary file 1 [file biomolecules-11-01695-s001.zip › biomolecules-1421580-supplementary/Supplementary data/FigureS6 - western blot quantification_v2.pdf]

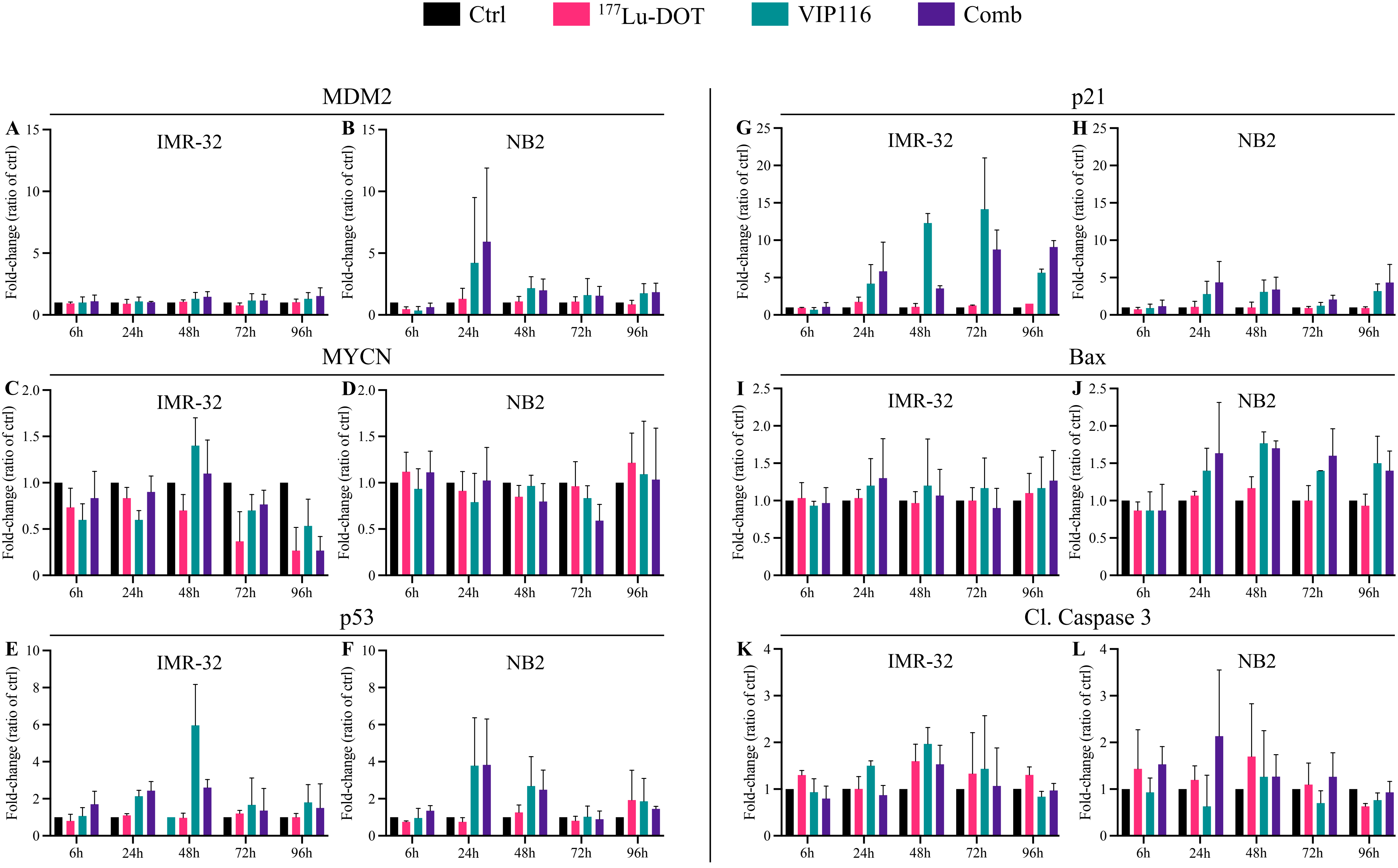

Figure S6: Quantification of western blots stained for (a-b) MDM2, (c-d) MYCN, (e-f) p53, (g-h) p21, (i-j) Bax and (k-l) cleaved caspase 3 (mean±SD, N≥3).
