# Supplementary material for: p53-Mediated Radiosensitization of 177Lu-DOTATATE in Neuroblastoma Tumor Spheroids
Source: Biomolecules. 2021 Nov 15;11(11):1695. doi: 10.3390/biom11111695 (PMC8615514; doi:10.3390/biom11111695)
Supplement: Supplementary file 1 [file biomolecules-11-01695-s001.zip › biomolecules-1421580-supplementary/Supplementary data/FigureS7-flow cytometry_v4.pdf]

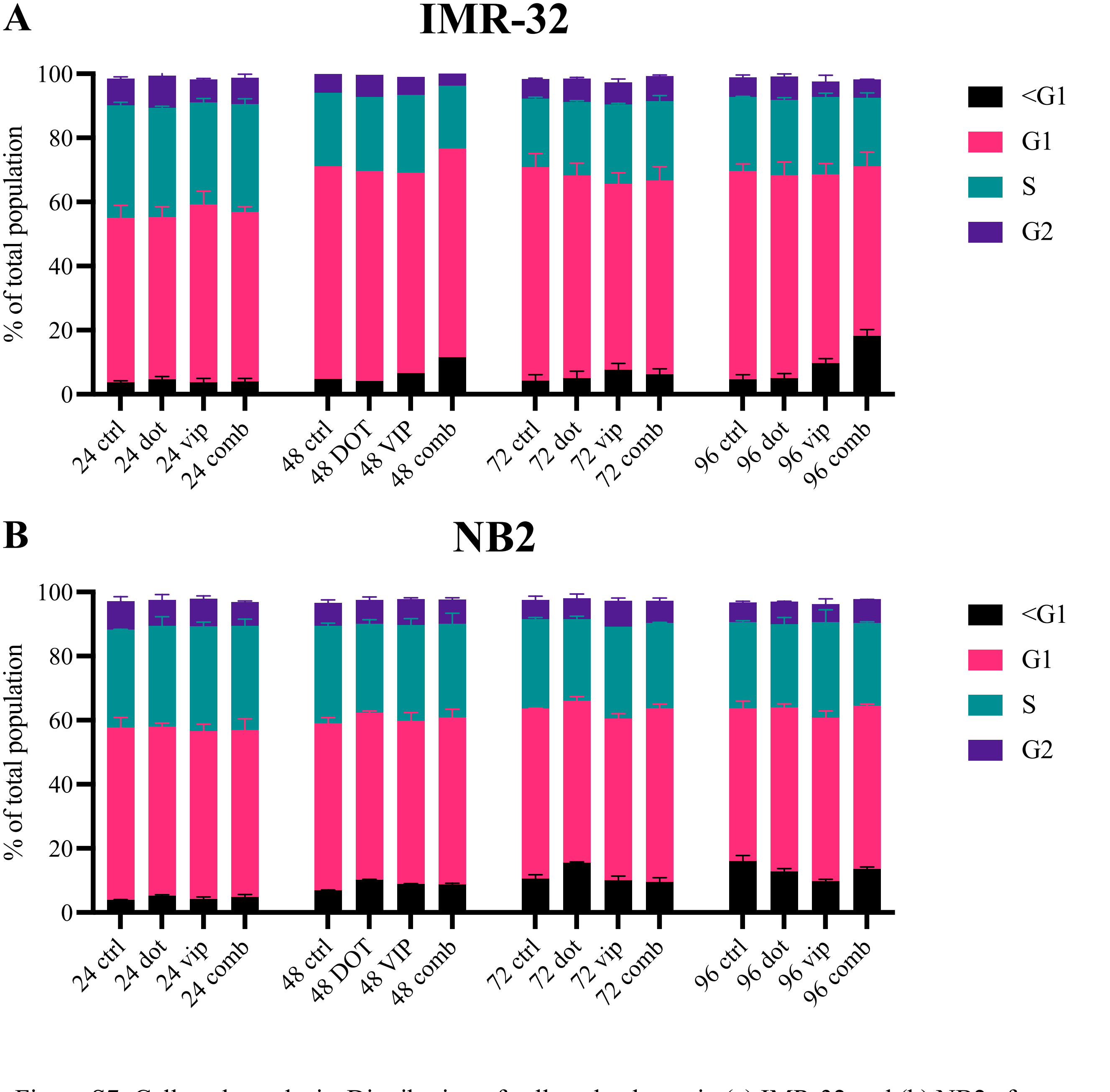

Figure S7: Cell cycle analysis. Distribution of cell cycle phases in (a) IMR-32 and (b) NB2 after treatment with ^{177}Lu-DOTATATE and/or VIP116. Pooled data from 2 experiments (except for IMR-32 at 48 hours, with only 1 replicate), presented as mean±SD.
