# Supplementary material for: p53-Mediated Radiosensitization of 177Lu-DOTATATE in Neuroblastoma Tumor Spheroids
Source: Biomolecules. 2021 Nov 15;11(11):1695. doi: 10.3390/biom11111695 (PMC8615514; doi:10.3390/biom11111695)
Supplement: Supplementary file 1 [file biomolecules-11-01695-s001.zip › biomolecules-1421580-supplementary/Supplementary data/Supplementary data.pdf]

Table S1. List of sequencing primers

Human primers		
**Exon**	**Direction**	**Sequence (5′-3′)**
2+3	Forward	CTGTCTCAGACACTGGCATGG
	Reverse	GGCAAGGGGGACTGTA
4	Forward	GGACTGACTTTCTGCTCTTGTCTTT
	Reverse	CAGAGATCACACATTAAGTGGGTAA
5	Forward	CTCTCTAGCTCGCTAGTGGGT
	Reverse	CGAAAAGTGTTTCTGTCATCCAAAT
6	Forward	GCCATGGCCATCTACAAGCA
	Reverse	TGGGGTTATAGGGAGGTCAAA
7	Forward	ACAGGTCTCCCCAAGG
	Reverse	AAACTGAGTGGGAGCAGTAAGGAGA
8+9	Forward	GGACAAGGGTGGTTGGGAGTAGA
	Reverse	CCCAATTGCAGGTAAAACAGTCAAG
10	Forward	CAGTTTCTACTAAATGCATGTTGCT
	Reverse	ATACACTGAGGCAAGAATGTGGTTA
11	Forward	CATCTTGATTTGAATTCCCGTTGT
	Reverse	CACCAGTGCAGGCCAACTTGTTCAG
**Murine primers**		
**Exon**	**Direction**	**Sequence (5′-3′)**
2	Forward	GCATCCATACAGTACACAATCTCT
	Reverse	CCAACACCATACCATGTTTGAACA
3+4	Forward	GAACTAACTCTCTGCTCTTGT
	Reverse	AGGTCACACGAAAGACAACTC
5	Forward	TTCCAGTACTCTCCTCCCCTCAA
	Reverse	AGGCTGCCAGTCCTAACCCCACAG
6	Forward	CATCTCCCGGCTTCTGACTTA
	Reverse	AGTCTAGGCTGGAGTCAACTG
7	Forward	GAGGTAGGGAGCGACTTCAC
	Reverse	GCTGGGGAAGAAACAGGCTAACCT
8+9	Forward	GGGCCCAGCTTTCTTACTGC
	Reverse	AGACAGAGGCAATAATGGGTACA
10	Forward	TGTCCAGTGCTTCCATCTCAC
	Reverse	TCACTACAAAGGCTGAGCTGG
11	Forward	AGCTCCCATCACTTCATCCCTC
	Reverse	CCTGGACTCAGGTGGGTACCT

Table S2. List of materials for western blot experiments

Article	Ref. no.	Company
SureLock Tandem Midi Gel Tank	STM1001	Thermo Fisher
SureLock Tandem Midi Blot Module	STM2001	Thermo Fisher
NuPAGE MOPS Running buffer	NP0001	Thermo Fisher
NuPAGE 4-12% Bis-Tris Midi gel	WG1403BOX	Thermo Fisher
Non-reducing loading sample buffer	LC2570	Thermo Fisher
NuPAGE sample reducing agent	NP0009	Thermo Fisher
iBright prestained protein ladder	LC5615	Thermo Fisher
PVDF membranes, 0.45 μm	STM2006	Thermo Fisher
NuPAGE transfer buffer	NP0006	Thermo Fisher
Blocking buffer	37565	Thermo Fisher
Bovine Serum Albumin	A7906	Sigma Aldrich
Pico PLUS Chemiluminescent substrate	34577	Thermo Fisher
Femto Chemiluminescent substrate	34094	Thermo Fisher

Table S3. p53 variants detected in the current study

Cell line	Exon number	Genomic description (hg 38)	Genomic description (GRCm39)	cDNA description*	Protein description	IARC ID	dbSNP	Effect
NB1, NB2, SKNAS	Exon 4	g.7676154G>C	n.a	c.215C>G	p.P72R	708	rs1042522	Validated SNP. Potentially functional polymorphism with reported significance in drug response. Benign, uncertain significance.
Neuro2a	Exon 4	n.a.	g.69478314C>T	c.379 C>T	p.T74T	n.a.	n.a.	Silent variant
Neuro2a	Exon 5	n.a.	g.69479331G>C	c.665 G>C	p.V170L	n.a.	n.a.	Hydrofobic to hydrofobic missense mutation in the DNA binding region

*According to IARC nomenclature for human TP53 variants, referring to the NM_000546.6 and alignment to NM011640.3 for the mouse lines N1e and N2a

Flow cytometry – cell cycle analysis – IMR-32

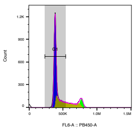

24 h ctrl1.fcs
Cell Cycle
Pseudocolor of FSC-H() vs. FL6-A()
14831

RMSD : 4.09
%G1 : 54.1
%S : 34.3
%G2 : 8.12
G1 Mean : 384691
G2 Mean : 7.73E5
G1 CV : 5.12
G2 CV : 4.24
% less G1 : 3.32
% greater G2 : 0.38

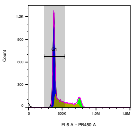

24h DOT.fcs
Cell Cycle
Pseudocolor of FSC-A() vs. FSC-H()
14651

RMSD : 4.05
%G1 : 53.0
%S : 33.4
%G2 : 9.49
G1 Mean : 378247
G2 Mean : 7.59E5
G1 CV : 5.10
G2 CV : 4.84
% less G1 : 3.94
% greater G2 : 0.14

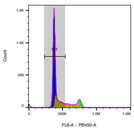

24h VIP.fcs
Cell Cycle
Pseudocolor of FSC-A() vs. FSC-H()
15090

RMSD : 3.79
%G1 : 58.4
%S : 30.8
%G2 : 7.45
G1 Mean : 375831
G2 Mean : 7.58E5
G1 CV : 4.92
G2 CV : 4.34
% less G1 : 2.99
% greater G2 : 0.24

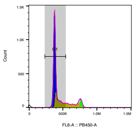

24h comb.fcs
Cell Cycle
Pseudocolor of FSC-A() vs. FSC-H()
14605

RMSD : 3.84
%G1 : 54.0
%S : 34.7
%G2 : 7.46
G1 Mean : 376995
G2 Mean : 7.64E5
G1 CV : 4.62
G2 CV : 3.99
% less G1 : 3.44
% greater G2 : 0.24

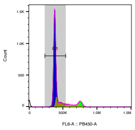

48h DOT.fcs
Cell Cycle
Pseudocolor of FSC-A() vs. FSC-H()
14068

RMSD : 5.36
%G1 : 65.5
%S : 23.0
%G2 : 6.89
G1 Mean : 379650
G2 Mean : 7.60E5
G1 CV : 5.05
G2 CV : 5.00
% less G1 : 4.24
% greater G2 : 0.050

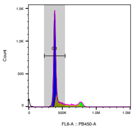

48h VIP.fcs
Cell Cycle
Pseudocolor of FSC-A() vs. FSC-H()
13901

RMSD : 5.72
%G1 : 62.5
%S : 24.3
%G2 : 5.64
G1 Mean : 387246
G2 Mean : 7.77E5
G1 CV : 4.76
G2 CV : 4.55
% less G1 : 6.63
% greater G2 : 0.32

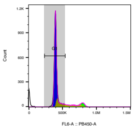

48h comb.fcs
Cell Cycle
Pseudocolor of FSC-A() vs. FSC-H()
11268

RMSD : 8.12
%G1 : 65.2
%S : 19.5
%G2 : 4.51
G1 Mean : 395787
G2 Mean : 8.03E5
G1 CV : 4.97
G2 CV : 4.51
% less G1 : 11.6
% greater G2 : -0.23

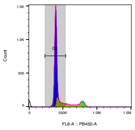

48h ctrl.fcs
Cell Cycle
Pseudocolor of FSC-A() vs. FSC-H()
14290

RMSD : 4.73
%G1 : 66.3
%S : 22.9
%G2 : 5.95
G1 Mean : 394845
G2 Mean : 7.95E5
G1 CV : 5.12
G2 CV : 4.42
% less G1 : 4.81
% greater G2 : 0.28

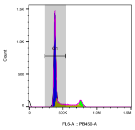

72h DOT.fcs
Cell Cycle
Pseudocolor of FSC-A() vs. FSC-H()
13437

RMSD : 5.17
%G1 : 65.9
%S : 22.5
%G2 : 7.25
G1 Mean : 381912
G2 Mean : 7.65E5
G1 CV : 4.85
G2 CV : 4.16
% less G1 : 3.54
% greater G2 : 0.92

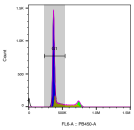

72h VIP.fcs
Cell Cycle
Pseudocolor of FSC-A() vs. FSC-H()
12777

RMSD : 6.02
%G1 : 60.5
%S : 25.0
%G2 : 6.33
G1 Mean : 370720
G2 Mean : 7.45E5
G1 CV : 4.31
G2 CV : 4.20
% less G1 : 6.19
% greater G2 : 0.93

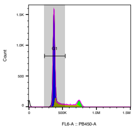

72h comb.fcs
Cell Cycle
Pseudocolor of FSC-A() vs. FSC-H()
14273

RMSD : 5.76
%G1 : 63.6
%S : 23.5
%G2 : 8.06
G1 Mean : 374079
G2 Mean : 7.48E5
G1 CV : 4.66
G2 CV : 4.74
% less G1 : 4.91
% greater G2 : 0.28

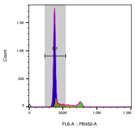

72h ctrl.fcs
Cell Cycle
Pseudocolor of FSC-A() vs. FSC-H()
13844

RMSD : 4.10
%G1 : 69.6
%S : 21.0
%G2 : 6.00
G1 Mean : 377148
G2 Mean : 7.61E5
G1 CV : 4.66
G2 CV : 4.07
% less G1 : 2.99
% greater G2 : 0.24

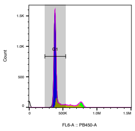

96h DOT.fcs
Cell Cycle
Pseudocolor of FSC-A() vs. FSC-H()
14644

RMSD : 5.54
%G1 : 66.2
%S : 23.0
%G2 : 6.61
G1 Mean : 382883
G2 Mean : 7.71E5
G1 CV : 4.95
G2 CV : 4.80
% less G1 : 4.12
% greater G2 : 0.28

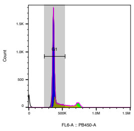

96h VIP.fcs
Cell Cycle
Pseudocolor of FSC-A() vs. FSC-H()
14330

RMSD : 8.87
%G1 : 61.3
%S : 23.3
%G2 : 6.22
G1 Mean : 369148
G2 Mean : 7.37E5
G1 CV : 4.24
G2 CV : 4.96
% less G1 : 8.77
% greater G2 : 0.22

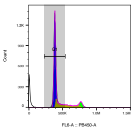

96h comb.fcs
Cell Cycle
Pseudocolor of FSC-A() vs. FSC-H()
14221

RMSD : 16.4
%G1 : 56.0
%S : 20.4
%G2 : 5.74
G1 Mean : 387235
G2 Mean : 7.73E5
G1 CV : 4.43
G2 CV : 4.09
% less G1 : 16.8
% greater G2 : 0.54

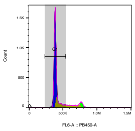

96h ctrl.fcs
Cell Cycle
Pseudocolor of FSC-A() vs. FSC-H()
14321

RMSD : 4.18
%G1 : 66.6
%S : 23.2
%G2 : 5.76
G1 Mean : 385413
G2 Mean : 7.73E5
G1 CV : 4.77
G2 CV : 4.09
% less G1 : 3.60
% greater G2 : 0.55

Flow cytometry – cell cycle analysis – NB2

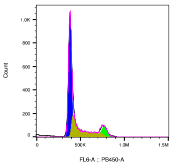

24h DOT.fcs
Cell Cycle
Pseudocolor of FSC-H() vs. FL6-A()
14560

RMSD : 3.71
%G1 : 53.6
%S : 29.4
%G2 : 9.21
G1 Mean : 382725
G2 Mean : 7.71E5
G1 CV : 6.16
G2 CV : 6.36
% less G1 : 5.00
% greater G2 : 1.46

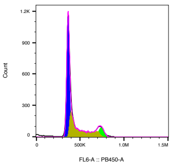

24h VIP.fcs
Cell Cycle
Pseudocolor of FSC-H() vs. FL6-A()
15806

RMSD : 4.29
%G1 : 50.7
%S : 33.7
%G2 : 8.00
G1 Mean : 365004
G2 Mean : 7.42E5
G1 CV : 5.86
G2 CV : 6.09
% less G1 : 4.69
% greater G2 : 1.57

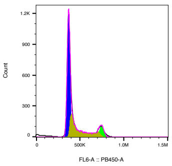

24h comb.fcs
Cell Cycle
Pseudocolor of FSC-H() vs. FL6-A()
15291

RMSD : 3.77
%G1 : 54.7
%S : 30.9
%G2 : 7.64
G1 Mean : 370700
G2 Mean : 7.48E5
G1 CV : 5.90
G2 CV : 4.84
% less G1 : 4.17
% greater G2 : 1.69

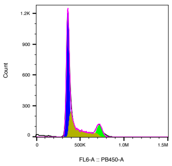

24h ctrl.fcs
Cell Cycle
Histogram* of FL6-A(null)
16196

RMSD : 3.75
%G1 : 51.6
%S : 30.6
%G2 : 9.84
G1 Mean : 359509
G2 Mean : 7.21E5
G1 CV : 5.96
G2 CV : 6.04
% less G1 : 4.00
% greater G2 : 2.60

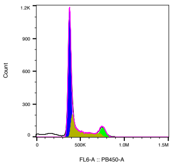

48h DOT.fcs
Cell Cycle
Pseudocolor of FSC-H() vs. FL6-A()
15054

RMSD : 4.96
%G1 : 52.6
%S : 26.6
%G2 : 8.16
G1 Mean : 377012
G2 Mean : 7.56E5
G1 CV : 5.78
G2 CV : 6.07
% less G1 : 10.0
% greater G2 : 1.56

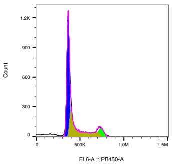

48h VIP.fcs
Cell Cycle
Pseudocolor of FSC-H() vs. FL6-A()
15388

RMSD : 5.00
%G1 : 49.1
%S : 31.3
%G2 : 7.84
G1 Mean : 364228
G2 Mean : 7.38E5
G1 CV : 5.40
G2 CV : 6.30
% less G1 : 8.69
% greater G2 : 1.64

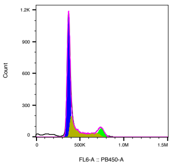

48h comb.fcs
Cell Cycle
Pseudocolor of FSC-H() vs. FL6-A()
15200

RMSD : 4.18
%G1 : 54.0
%S : 26.8
%G2 : 7.94
G1 Mean : 370309
G2 Mean : 7.44E5
G1 CV : 5.85
G2 CV : 6.11
% less G1 : 8.42
% greater G2 : 1.50

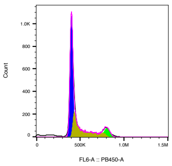

48h ctrl.fcs
Cell Cycle
Pseudocolor of FSC-H() vs. FL6-A()
14696

RMSD : 4.23
%G1 : 51.1
%S : 30.9
%G2 : 7.83
G1 Mean : 4.01E5
G2 Mean : 8.06E5
G1 CV : 5.58
G2 CV : 5.94
% less G1 : 6.68
% greater G2 : 1.81

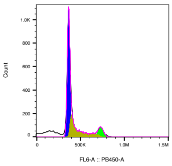

72h DOT.fcs
Cell Cycle
Pseudocolor of FSC-H() vs. FL6-A()
14288

RMSD : 5.83
%G1 : 49.8
%S : 24.7
%G2 : 7.51
G1 Mean : 366628
G2 Mean : 7.33E5
G1 CV : 5.70
G2 CV : 5.78
% less G1 : 15.3
% greater G2 : 1.56

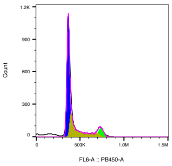

72h VIP.fcs
Cell Cycle
Pseudocolor of FSC-H() vs. FL6-A()
14575

RMSD : 5.13
%G1 : 51.6
%S : 28.6
%G2 : 7.57
G1 Mean : 366602
G2 Mean : 7.37E5
G1 CV : 5.56
G2 CV : 5.63
% less G1 : 9.06
% greater G2 : 1.70

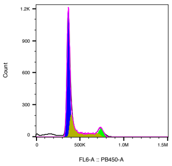

72h comb.fcs
Cell Cycle
Pseudocolor of FSC-H() vs. FL6-A()
14622

RMSD : 5.21
%G1 : 55.1
%S : 26.4
%G2 : 6.45
G1 Mean : 367630
G2 Mean : 7.41E5
G1 CV : 5.73
G2 CV : 5.21
% less G1 : 8.61
% greater G2 : 1.65

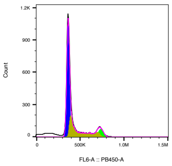

72h ctrl.fcs
Cell Cycle
Pseudocolor of FSC-H() vs. FL6-A()
15151

RMSD : 5.27
%G1 : 53.2
%S : 28.3
%G2 : 6.80
G1 Mean : 363107
G2 Mean : 7.37E5
G1 CV : 5.95
G2 CV : 5.69
% less G1 : 9.64
% greater G2 : 1.25

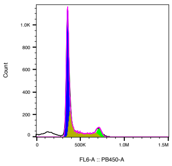

96h DOT.fcs
Cell Cycle
Pseudocolor of FSC-H() vs. FL6-A()
13500

RMSD : 5.46
%G1 : 50.5
%S : 27.4
%G2 : 6.86
G1 Mean : 354374
G2 Mean : 7.15E5
G1 CV : 5.30
G2 CV : 5.45
% less G1 : 12.0
% greater G2 : 1.66

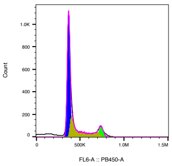

96h VIP.fcs
Cell Cycle
Pseudocolor of FSC-H() vs. FL6-A()
14252

RMSD : 4.71
%G1 : 52.4
%S : 27.2
%G2 : 6.72
G1 Mean : 370852
G2 Mean : 7.41E5
G1 CV : 5.59
G2 CV : 4.59
% less G1 : 9.46
% greater G2 : 2.67

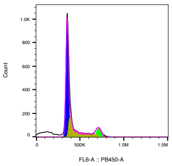

96h comb.fcs
Cell Cycle
Pseudocolor of FSC-H() vs. FL6-A()
13533

RMSD : 6.07
%G1 : 50.6
%S : 25.6
%G2 : 7.32
G1 Mean : 355965
G2 Mean : 7.17E5
G1 CV : 5.56
G2 CV : 5.55
% less G1 : 13.0
% greater G2 : 1.62

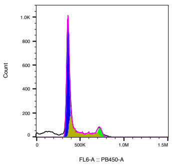

96h ctrl.fcs
Cell Cycle
Pseudocolor of FSC-H() vs. FL6-A()
13190

RMSD : 5.63
%G1 : 49.3
%S : 26.3
%G2 : 5.96
G1 Mean : 361037
G2 Mean : 7.26E5
G1 CV : 5.59
G2 CV : 4.56
% less G1 : 14.8
% greater G2 : 2.41
